# Nuclear overexpression of the overexpressed in lung cancer 1 predicts worse prognosis in gastric adenocarcinoma

**DOI:** 10.18632/oncotarget.14217

**Published:** 2016-12-26

**Authors:** Jue Wang, Hongchang Shen, Guobin Fu, Dandan Zhao, Weibo Wang

**Affiliations:** ^1^ Department of Breast and Thyroid Surgery, Shandong Provincial Hospital Affiliated to Shandong University, Jinan, Shandong, China; ^2^ Department of Chemotherapy, Shandong Provincial Hospital Affiliated to Shandong University, Jinan, Shandong, China

**Keywords:** gastric adenocarcinoma, OLC1, IHC, prognosis, subcellular expression

## Abstract

We have performed this retrospective study to elucidate whether elevated expression of the overexpressed in lung cancer 1 (OLC1) was related to the clinicopathological parameters and prognosis of patients with gastric adenocarcinoma. Additionally, different effects of various subcellular OLC1 expression on gastric adeno-carcinogenesis were focused on in our study. Both overall and subcellular expression of OLC1 was evaluated by immunohistochemistry(IHC) via tissue microarrays from total 393 samples. The Kaplan-Meier method and Cox's proportional hazard model were exerted to further explore the correlation between OLC1 and prognosis. Total overexpression of OLC1 was significantly associated with stage (*P* = 0.004) and differentiation (*P* = 0.009), and only the strong total expression could predict a poor prognosis (HR = 1.31, *P* = 0.04). There were significant associations found between nuclear overexpression and tumor invasion depth(*P* = 0.002), lymph node (*P* < 0.001), stage (*P* = 0.004), differentiation (*P* < 0.001) and smoking history (*P* = 0.045). Furthermore, over-expressed nuclear OLC1 protein could be an independent risk factor for gastric adenocarcinoma (univariate: HR = 1.43, *P* = 0.003; multivariate: HR = 1.39, *P* = 0.011). In general, both total and nuclear overexpression of OLC1 could be the signs of gastric adeno-carcinogenesis, which might be served as the biomarkers for diagnosis at an early stage, even at the onset of tumorigenesis. Rather than the total expression, nuclear overexpression of OLC1 was correlated with most clinicopathological parameters and could predict a poor overall survival as an independent factor for prognosis, which made it a more effective and sensitive biomarker for gastric adenocarcinoma.

## INTRODUCTION

As the fifth worldwide malignancy, gastric cancer is the third leading cause of cancer mortality in both sexes. Approximately, one million new cases are diagnosed with gastric carcinoma per year since 2012, causing more than 700,000 deaths annually [1]. Nearly half of the new cases are detected in Asia, mostly from China [2]. Among gastric cancer, almost all death was result from gastric adenocarcinoma [3]. Despite of the development of diagnostic methods, such as the prevalence and advance of endoscopic screening, diagnosis of the vast majority of gastric adenocarcinoma is still delayed, which leads to missing the best time of treatment. Usually locally advanced or even metastatic disease are developed along the way, which accounts for the low rate of 5-year overall survival. Thus, it is urgent and essential to discover effective biomarkers to help diagnose at an early stage, predict prognosis and further promote advance of treatment for patients with gastric carcinoma.

The overexpressed in lung cancer 1 (OLC1) was originally identified by Yuan et al in human squamous cell lung tumors. According to their study, the overexpression of OLC1 protein was related to lung tumorigenesis and associated with patients’ smoking history [4]. Recent studies have revealed that OLC1 might be linked to activation of both mitogen-activated protein kinase (MAPK) and nuclear factor-κB (NF-κB) signaling pathway [4, 5] Besides high expression in pulmonary pre-malignant lesions and the most common pathological types of lung carcinoma, OLC1 protein overexpression was detected in a range of human cancers, such as epithelial ovarian cancer, esophageal squamous cell cancer, breast cancer and colorectal cancer [6–9]. Based on their discoveries, elevated expression of OLC1 protein promoted oncogenesis and predicted a poor prognosis.

The OLC1 gene was named after Increase Sodium Tolerance 1(IST1) gene as well, which was identified in non-lethal yeast mutants. On the basis of different subcellular localizations and recognition of certain protein-binding partners, IST1 regulates the disassembly of endosomal sorting complexes in the yeast [10, 11]. As the gene product of OLC1/IST1 is highly conserved from yeast to humans, the influences of diverse subcellular localizations of OLC1 protein may vary in tumorigenesis.

Given all current studies, we wondered if OLC1 is a potential marker for gastric cancer, particularly for gastric adenocarcinoma. Our study was intended to reveal the correlation between OLC1 and clinicopathologic parameters and prognosis in patients with gastric tumors. Additionally, considering only total protein expression was well studied in human cancers, effects of different subcellular OLC1 expression were ignored in the past. Thus, it was another focus of our study to elucidate the influences of different subcellular expression of OLC1 on gastric cancer, especially on gastric adenocarcinoma.

## RESULTS

### Expression levels of OLC1 in the whole study population

Among the benign gastric disease, which comprised gastritis and ulcer in our study, no high expression of OLC1 protein was detected both in the whole cell and nucleus. When considering the atypical hyperplasia patients, the rate of moderate and high total expression was 75% and 25% respectively, and only low level of nuclear expression was found. However, as for the specimens from gastric malignancies, almost 35% of them were discovered with high total expression and half of them were with moderate expression. Comparing with the samples from benign gastric disease and atypical hyperplasia, the proportion of moderate nuclear expression was approximately 50% and that of high was nearly 25% in gastric malignancies. Expression levels of OLC1 immunostaining were observed to gradually elevate from benign gastric disease to gastric tumors, which was verified not only by total expression, but also by nuclear expression (Table 1).

**Table 1:** The relationship between OLC1 protein expression and clinicopathological characteristics of the whole study population

		Total OLC1 Expression				Cytoplasmic OLC1 Expression				Membranous OLC1 Expression				Nuclear OLC1 Expression			
	No.	Low	Moderate	High	*P*	Low	Moderate	High	*P*	Low	Moderate	High	*P*	Low	Moderate	High	*P*
**Gender**																	
Male	292	42	148	102	0.7	77	175	40	0.255	143	107	42	0.887	81	143	68	0.751
Female	101	18	50	33		33	51	17		52	36	13		32	47	22	
Age																	
< 60	179	26	91	62	0.932	52	100	27	0.834	83	66	30	0.284	49	85	45	0.61
> = 60	214	34	107	73		58	126	30		112	77	25		64	105	45	
**General**																	
Benign	6	4	2	0	**0.009**	4	2	0	**0.189**	6	0	0	**0.065**	5	1	0	**0.001**
Atypical Hyperplasia	4	0	3	1		2	2	0		1	3	0		4	0	0	
Malignant	383	56	193	134		104	222	57		188	140	55		104	189	90	
**Tumor Pathological type**																	
Gastrointestinal Stromal Tumors(GIST)	13	4	7	2	**0.032**	6	6	1	0.081	9	4	0	0.598	2	4	7	0.245
Gastric signet ring cell carcinoma	12	1	4	7		2	7	3		4	7	1		3	8	1	
Gastric squamous cell carcinoma	4	0	2	2		0	4	0		2	1	1		0	3	1	
Gastric neuroendocrine tumor	3	2	0	1		2	0	1		2	0	1		1	2	0	
Gastric lymphoma	1	1	0	0		1	0	0		1	0	0		0	1	0	
Gastric sarcoma	1	0	0	1		0	0	1		1	0	0		1	0	0	
Gastric Adenocarcinoma	349	48	180	121		93	205	51		169	128	52		97	171	81	

In gastric tumors, the total expression level of OLC1 varied in different pathologic types. Both gastric lymphoma (100%) and neuroendocrine tumor (66.7%) were detected with a higher rate of low total expression. The higher proportion of moderate total expression was found in GIST (53.8%), gastric squamous cell carcinoma (50%) and gastric adenocarcinoma (51.6%). The highest ratio of high total expression existed in the subgroup of gastric signet ring cell carcinoma (58.3%). Based on the results above, we found that total expression levels of OLC1 in various pathologic types of gastric tumors were statistically significant (*P* = 0.032, Table 1). Therefore, it was implied that the impacts of OLC1 tumorigenesis effects might alter on different pathologic types of gastric carcinomas.

### Correlations between OLC1 expression levels and clinicopathological parameters in population with gastric adenocarcinoma

Since there was a significant difference of OLC1 total expression among various pathologic types of gastric cancer, it was necessary to investigate the correlation between OLC1 expression levels and clinicopathological parameters in each specific pathologic type, which was gastric adenocarcinoma here.

**Figure 1:**
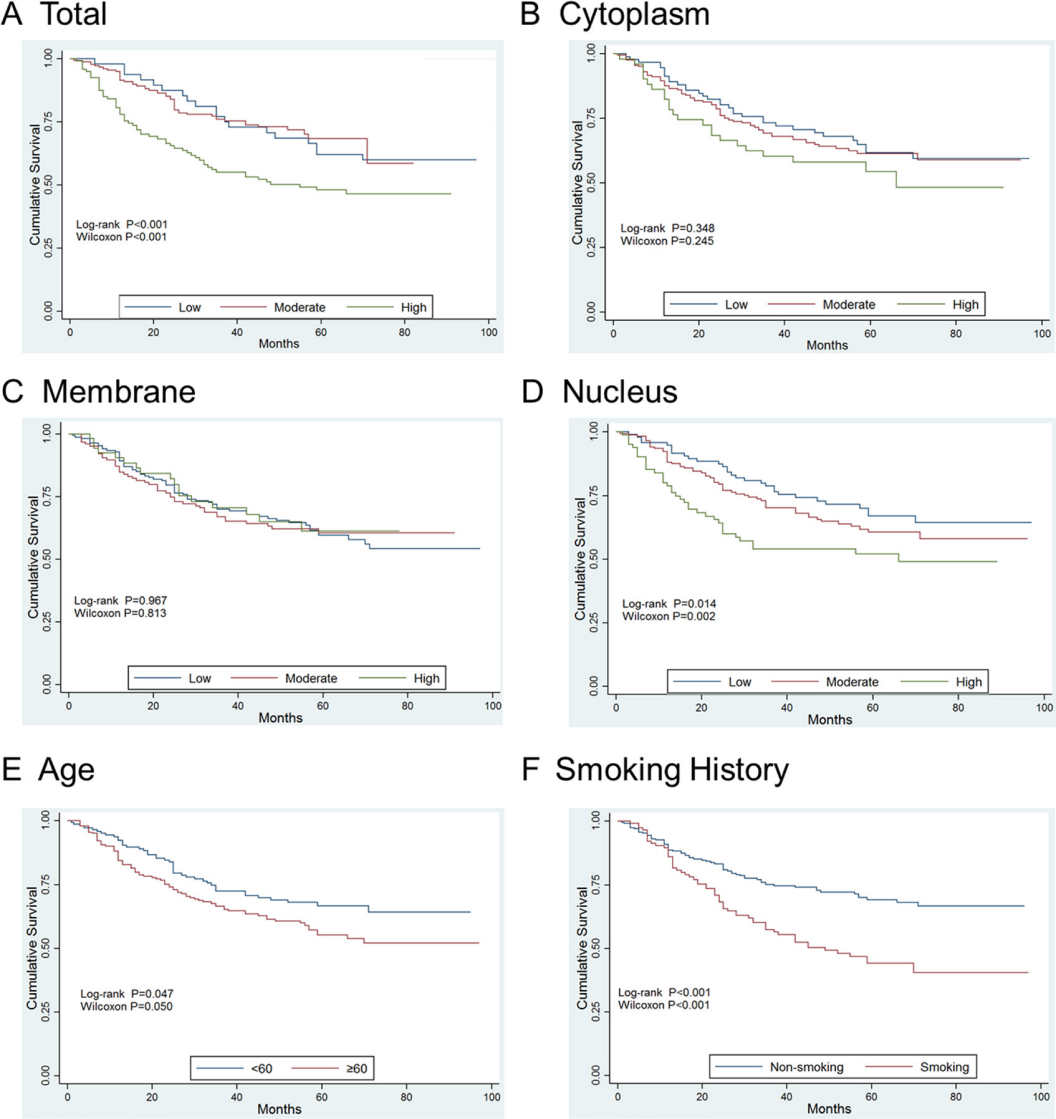
Representative examples showing different level of OLC1 expression (**A**) Low total expression; (**B**) Moderate total expression; (**C**) High total expression; (**D**) Low nuclear expression; (**E**) Moderate nuclear expression; (**F**) High nuclear expression.

According to the immunostaining evaluation, in general, OLC1 lowly (Figure 1A), moderately (Figure 1B) and highly (Figure 1C) expressed in 48(13.7%), 180(51.6%) and 121(34.7%) of the total 349 gastric adenocarcinoma patients respectively. Total overexpression of OLC1 was significantly associated with stage (*P* = 0.004, Table 2) and gastric adenocarcinoma differentiation (*P* = 0.009, Table 2).

**Table 2:** The relationship between OLC1 protein expression and gastric adenocarcinoma clinicopathological characteristics

		Total OLC1 Expression				Cytoplasmic OLC1 Expression				Membranous OLC1 Expression				Nuclear OLC1 Expression			
	No.	Low	Moderate	High	*P*	Low	Moderate	High	*P*	Low	Moderate	High	*P*	Low	Moderate	High	*P*
**Tumor (Invasion Depth)**																	
Tis^1^	7	2	4	1	0.25	1	5	1	0.879	3	3	1	0.966	5	2	0	**0.002**
1	51	7	24	20		13	31	7		26	17	8		18	16	17	
2	38	9	17	12		13	21	4		22	12	4		9	18	11	
3	44	8	18	18		14	22	8		20	18	6		11	17	16	
4	209	22	117	70		52	126	31		98	78	33		54	118	37	
**Lymph Node Grade**																	
0	128	16	73	39	0.076	39	77	12	0.297	68	42	18	0.603	41	56	31	**< 0.001**
1	51	10	16	25		13	28	10		28	17	6		14	27	10	
2	61	10	33	18		16	32	13		27	23	11		12	36	13	
3	109	12	58	39		25	68	16		46	46	17		30	52	27	
**Metastasis**																	
No	342	46	178	118	0.366	91	201	50	0.993	166	125	51	0.941	93	169	80	0.216
Yes	7	2	2	3		2	4	1		3	3	1		4	2	1	
**Stage**																	
0	7	1	5	1	**0.004**	1	5	1	0.129	4	2	1	0.904	4	2	1	**0.004**
I	71	15	31	25		20	40	11		40	21	10		23	27	21	
II	71	12	47	12		27	41	3		35	27	9		20	42	9	
III	193	18	95	80		43	115	35		87	75	31		46	97	50	
IV	7	2	2	3		2	4	1		3	3	1		4	3	0	
Differentiation*																	
Well	7	0	6	1	**0.009**	1	6	0	0.6	6	0	1	**< 0.001**	4	0	3	**< 0.001**
Moderate	102	18	54	30		27	61	14		70	27	5		32	43	27	
Moderate-Poor	72	10	45	17		16	42	14		42	26	4		11	41	20	
Poor	153	15	69	69		44	89	20		43	70	40		41	84	28	
**Smoking History**																	
No	234	33	124	77	0.614	68	139	27	**0.045**	111	84	39	0.668	61	126	51	0.045
Yes	115	15	56	44		25	66	24		58	42	15		35	45	31	

When considering the relation between OLC1 expression and gastric adenocarcinoma differentiation, gastric mucinous adenocarcinoma was ruled out as they didn't have differentiation description.

Abbreviation: ^1^Tis: Tumor *in situ*

OLC1 expression was detected in the nucleus lowly (Figure 1D), moderately (Figure 1E) and highly (Figure 1F) in 97 (27.8%), 171 (45.1%) and 81 (23.2%) specimens respectively. Significant correlations were verified between the elevated nuclear expression and tumor invasion depth (*P* = 0.002, Table 2), lymph node grade (*P* < 0.001, Table 2), stage (*P* = 0.004, Table 2), differentiation (*P* < 0.001, Table 2) and smoking history (*P* = 0.045, Table 2).

Although there were no significant correlations between the cytoplasmic and membranous overexpression and clinicopathological characteristics of gastric adenocarcinoma, the cytoplasmic overexpression was related to smoking history significantly (*P* = 0.045, Table 2).

Based on all the results above, nuclear overexpression of OLC1, rather than total overexpression, seemed to be more closely correlated with clinicopathologic parameters of gastric adenocarcinoma. Therefore, nuclear expression of OLC1 might intend to play pivotal roles in gastric adeno-carcinogenesis and even the prognosis.

### Prognostic significance of OLC1 overexpression in population with gastric adenocarcinoma

**Figure 2:**
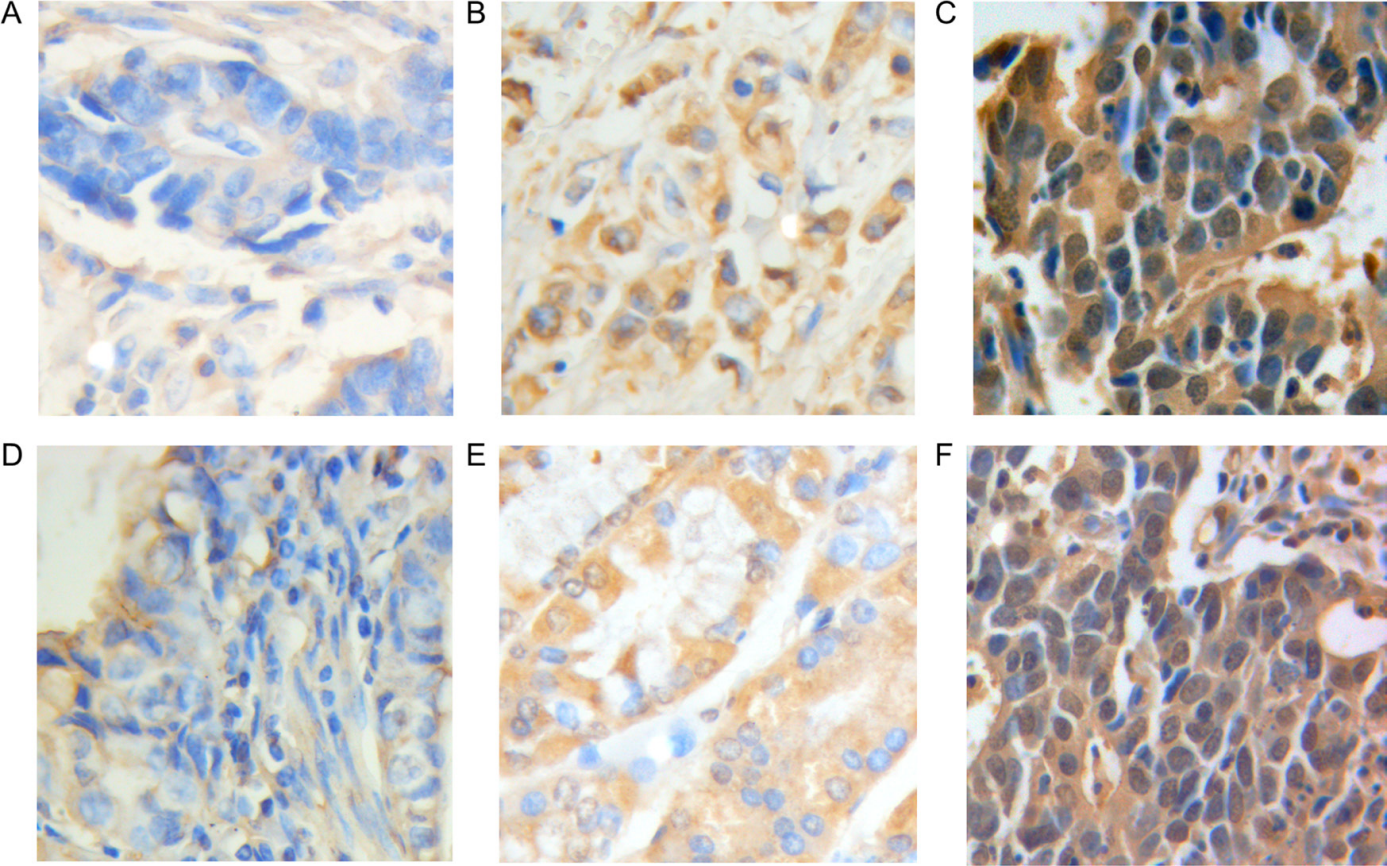
Kaplan–Meier curves for overall survival of prognosis in 349 patients with gastric adenocarcinoma (**A**) Total OLC1 expression; (**B**) Cytoplasmic OLC1 expression; (**C**) Membranous OLC1 expression; (**D**) Nuclear OLC1 expression; (**E**) Age; (**F**) Smoking.

To investigate the prognostic influence of OLC1 on gastric adenocarcinoma, we performed the Kaplan-Meier survival analysis along with both the log-rank and the Wilcoxon test. According to the Kaplan-Meier curves, influences of OLC1 total expression on prognosis were relatively complicated and vague. Despite the statistically significance found by both log-rank test (*P* < 0.001, Figure 2A) and Wilcoxon test (*P* < 0.001, Figure 2A), only strong total expression predicted a worse overall survival (OS) when comparing with low expression level. No significant difference was identified from the curves between the moderate expression level and the low level. On the other hand, it was revealed that nuclear overexpression was significantly related to a poor prognosis (Log-rank, *P* = 0.014; Wilcoxon, *P* = 0.002; Figure 2D), which was more clear and definite. There were no significant associations found between cytoplasmic (Log-rank, *P* = 0.348; Wilcoxon, *P* = 0.245; Figure 2B) and membranous (Log-rank, *P* = 0.967; Wilcoxon, *P* = 0.813; Figure 2C) overexpression and prognosis. Therefore, rather than total OLC1 overexpression, the nuclear overexpression was a better marker for gastric adenocarcinoma prognosis. In addition, old age (≥ 60, Log-rank, *P* = 0.047; Wilcoxon, *P* = 0.050; Figure 2E) and smoking history (Log-rank, *P* < 0.001; Wilcoxon, Wilcoxon, *P* < 0.001; Figure 2F) also predicted a worse OS in our study population.

To verify if OLC1 could be used as an independent risk factor for prognosis, common clinicopathological factors and OLC1 protein levels were assessed by Cox's univariate and multivariate hazard regression model (Table 3). It was indicated that tumor invasion depth [hazard ratio (HR) = 1.43, *P* < 0.001], lymph node involvement (HR = 1.71, *P* < 0.001), metastasis (HR = 5.61, *P* < 0.001), stage (HR = 2.50, *P* < 0.001), old age (≥ 60, HR = 1.43, *P* = 0.05), smoking history (HR = 2.07, *P* < 0.001) and nuclear overexpression (HR = 1.43, *P* = 0.003) were significantly correlated with a poor OS. As for the total expression, only the high expression level predicted a worse prognosis (HR = 1.31, *P* = 0.04). After exerting the multivariate analysis, the nuclear overexpression of OLC1 (HR = 1.39, *P* = 0.011), along with old age (≥ 60, HR = 1.55, *P* = 0.019), smoking history (HR = 2.07, *P* < 0.001), tumor invasion depth (HR = 1.42, *P* = 0.045), lymph node grade (HR = 1.48, *P* = 0.002) and metastasis (HR = 4.71, *P* = 0.005) could be independent prognostic factors for OS of gastric adenocarcinoma patients.

**Table 3:** Univariate and multivariate cox proportional hazards modeling analysis of 349 patients with gastric adenocarcinoma

	Univariate Hazard Ratio (95% CI)	*P*	Multivariate Hazard Ratio (95% CI)	*P*
**Age**				
< 60	Ref			
> = 60	1.43 (1.00,2.06)	**0.05**	1.55 (1.07,2.24)	**0.019**
**Tumor (Invasion Depth)**				
Tis	Ref			
1	1.72 (1.41,2.09)	< 0.001	1.42 (1.01,2.00)	**0.045**
2				
3				
4				
**Lymph Node Grade**				
0	Ref			
1	1.71(1.47,1.99)	< 0.001	1.48 (1.16,1.91)	**0.002**
2				
3				
**Metastasis**				
No	Ref			
Yes	5.61(2.61,12.08)	< 0.001	4.71 (1.60,13.85)	**0.005**
**Stage**				
0	Ref	< 0.001		
I	2.50 (1.91,3.28)		0.95 (0.51,1.80)	0.882
II				
III				
IV				
**OLC Total Expression**				
Low	Ref			
Moderate	0.89 (0.52,1.53)	0.68	1.06 (0.46,2.46)	0.88
High	1.31 (1.01,1.70)	0.04	1.12 (0.86,1.46)	0.41
**OLC Cytoplasmic Expression**				
Low	Ref			
Moderate	1.20 (0.91,1.57)	0.19	0.99 (0.74,1.31)	0.947
High				
**OLC Membranous Expression**				
Low	Ref			
Moderate	0.95 (0.74,1.21)	0.67	0.90 (0.70,1.16)	0.43
High				
**OLC Nuclear Expression**				
Low	Ref			
Moderate	1.43 (1.13,1.83)	**0.003**	1.39 (1.08,1.80)	**0.011**
High				
**Smoking History**				
No	Ref			
Yes	2.07 (1.46,2.94)	**< 0.001**	2.07 (1.45,2.97)	**< 0.001**

## DISCUSSION

It is well known that OLC1 exerts crucial effects on tumorigenesis of human lung and can be a potential diagnostic and therapeutic target for other human cancers [4, 6–9]. Yuan et al. has found that OLC1 was associated with activation of NF-κB signaling pathway [4]. Constitutive activation of NF-κB was detected in gastric tumors before and is closely intertwined with cancer growth and metastasis [12–14]. The activation of NF-κB is an early event during tumorigenesis, which can regulate expression of multiple genes involved in carcinoma formation, progression and invasion thereafter [15–17]. Thus, OLC1 tumorigenesis effects may be realized by activating NF-κB signaling pathway. Importantly, the expression level of OLC1 may alter at the very early stage of tumor formation, even at the onset of oncogenesis. Based on a large prospective cohort in our study, proportion of both total and nuclear OLC1 overexpression gradually elevated from benign disease, atypical hyperplasia to malignancies. It is implied that both total and nuclear OLC1 overexpression could be served as early warning signs of gastric malignant transformation in the future, which may help to diagnose gastric tumors at an early stage and to achieve the best timing for treatment.

With respect to gastric malignancies, there was a significantly difference of total expression when comparing various pathological types in our study. It was indicated that the impacts of OLC1 tumorigenesis effects differed on diverse pathological types. To clarify the correlation between OLC1 and clinicopathological parameters, we should study the associations in each certain pathological type of gastric carcinoma, instead of mixing them together.

As for gastric adenocarcinoma, the most prevalent and most lethal type of stomach tumors, the total overexpression of OLC1 was correlated with late stage and low differentiation. Previous studies have demonstrated that in lung cancer cells and tissues, the strong expression of OLC1 is highly related to smoking history and may result from suppression of ubiquitin-dependent degradation caused by cigarettes [4, 18]. Similar results existed in our study. Smoking history was an independent prognostic factor in gastric adenocarcinoma and a significant association was found between the expression of OLC1 and smoking history. However, the association was verified only by cytoplasmic and nuclear expression, instead of total OLC1 expression. Nuclear overexpression was not only correlated with most clinicopathological parameters of gastric adenocarcinoma, including tumor invasion depth, lymph node grade, stage, differentiation, old age and smoking history, but it could be served as an independent prognostic biomarker for gastric adenocarcinoma. Consequently, rather than the total expression, the nuclear OLC1 intends to be a more effective and efficient prognostic marker for gastric adenocarcinoma.

This is the first time to investigate the correlation between OLC1 and clinicopathologic parameters, as well as prognosis in patients with gastric adenocarcinoma. Additionally, it is the first time to reveal the different influences of various subcellular localizations of OLC1 on human cancers. According to our study, we may propose a hypothesis that cigarettes are able to suppress the degradation of OLC1 protein located in the nucleus, leading to the activation of NF-κB signaling pathway, finally resulting in gastric adeno-carcinogenesis. Further experiments need performing to elucidate the underlying oncogenesis mechanism. OLC1, especially that localized in the nucleus, has a great chance to be a novel treatment target for gastric adenocarcinoma and even to help prevent gastric adenocarcinoma at the onset of tumor formation.

Some limitations existed in our study as well. The size of benign gastric disease samples was a little insufficient because currently fewer and fewer surgeries for gastritis and ulcer are needed. With the small sample size of uncommon pathological types, we could not investigate the oncogenic effects of OLC1 on those types. As there were no differentiation pathologic reports on gastric mucinous adenocarcinoma, we could not get a more comprehensive conclusion of the correlation between OLC1 and adenocarcinoma differentiation. Additionally, it was too difficult to obtain accurate data for drinking and gastritis history and therefore, these two pivotal predispositions have been ruled out of our study. To improve these aspects, we will make progress in our follow-up records with more details in the future.

In conclusion, it is indicated that both total and nuclear OLC1 overexpression may be served as clinically-relevant indicators for early diagnosis. Furthermore, nuclear overexpression of OLC1 can predict a worse prognosis in patients with gastric adenocarcinoma. We hope our study can intrigue interests of oncologists to elucidate the underlying tumorigenesis mechanism of OLC1 and stress the emphasis on the importance of subcellular localizations of certain protein, which helps make progress in early diagnosing and developing individual accurate treatment for gastric adenocarcinoma patients.

## MATERIALS AND METHODS

### Patients and tumor samples

Immunohistochemical studies were performed on formalin-fixed paraffin-embedded tissues from patients who received gastric surgery from 2005 to 2010 in Shandong Provincial Hospital affiliated to Shandong University. This study totally involved 393 patients with a detailed long-term follow-up record. Patients who received standard treatment for gastric tumors were included in our study. Multiple clinicopathologic factors, including age at diagnosis, sex, major tumor characteristics and clinical follow-up data were obtained by medical record review. Patients were followed postoperatively every 3 months at the first year, then every 6 months during the second year to the fifth year and per year after 5 year. The vast majority of samples were from gastric tumors, 6 were from gastric benign disease, including inflammation and ulcer, and 4 were from atypical hyperplasia. Most of the malignant samples were from patients with gastric adenocarcinoma (*n* = 349). Besides, samples comprised other pathological types of gastric carcinomas, including gastrointestinal stromal tumor (GIST, *n* = 13), gastric signet ring cell carcinoma (*n* = 12), gastric squamous cell carcinoma (*n* = 4), gastric neuroendocrine tumor (*n* = 3), gastric lymphoma (*n* = 1) and sarcoma (*n* = 1). The median age of patients was 60 years old (range 23–86). The median clinical follow-up duration was 45 months, with an interval from 1 to 97 months.

### Tissue microarrays (TMA)

Tissue Microarray (TMA) construction was performed as described before by Richter et al. [19]. Representative carcinoma regions were defined by a hematoxylin and eosin-stained section from each paraffin block. The diameter of tissue cylinders is 0.6 mm^2^. These tissue cylinders were punched from paraffin blocks. Five-μm sections were cut from the identified TMA blocks and transferred to adhesive slides using the “paraffin-tape-transfer-system”.

### Immunohistochemistry (IHC)

TMA sections were deparaffinized with xylene, rehydrated through graded ethanol, rinsed in phosphate-buffered saline (PBS), exerted antigen retrieval and incubated with the primary antibody as described before [20]. Antigen retrieval was performed in 0.01 M sodium citrate (pH = 6.0) with heating in a pressure cooker. To quench endogenous peroxidase activity, the TMA slide was treated with 0.3% H_2_O_2_ for 30 minutes at room temperature. After treated with non-imunone serum albumin to block the non-specific binding, the slides were then incubated with primary antibody at 4°C overnight. This study used rabbit polyclonal anti-IST1 antibody (ab124368, Abcam Inc., Cambridge, MA, USA) as the primary antibody with 1:100 dilution. Then the second antibody from SP reagent kit (Zhongshan Goldenbridge Biotechnology Co., Beijing, China) was exerted to incubate the TMA sections for 1 hour at room temperature, followed by further incubation with streptavidin-horseradish peroxidase complex. Staining with 3,3′-diaminobenzidine kit (DAB; Zhongshan Goldenbridge Biotechnology Co.), TMA sections were counter-stained with hematoxylin and evaluated.

### Evaluation of immunostaining

The evaluation of immunostaining of TMC tissues was performed blindly by three independent observers (Jue Wang, Hongchang Shen and Dandan Zhao) and a consensus agreement was achieved. The scoring criteria of total OLC1 protein expression were as described before, considering both the percentage of positive cells and the staining intensity [21]. Additionally, the immunostaining of subcellular expression of OLC1 protein was assessed respectively. The principles of scoring both cytoplasmic and nuclear expression were the same as those used for evaluating total expression. As for the assessment of membranous expression, only the percentage of positive cells was taken into account. After calculation of staining index, the evaluation was defined as the level of low, moderate and high. The negative expression was categorized into the subgroup of low level expression.

### Ethics statements

All enrolled patients have given their informed consent. Ethical approval for this study was achieved from Ethical and Scientific Committees of Shandong Provincial Hospital affiliated to Shandong University.

### Statistical analysis

The chi-square test was performed to testify the correlation between the OLC1 expression and clinicopathologic parameters of gastric cancer. OS was used as the indicator of prognosis of gastric adenocarcinoma in our study. It was defined as the time from surgery until the date of death or the most recent follow-up. Overall survival curves were generated according to the Kaplan-Meier method, which verified by both the Wilcoxon test and the log-rank test. Cox's proportional hazard model was exerted for the univariate and multivariate analysis of prognostic values. Statistical significance was considered at *P* ≤ 0.05 two-tailed level throughout. All statistical analysis was accomplished by STATA 14.0 for Windows.
